# Traumatic Liver Injury With Delayed Bleeding After Extracorporeal Cardiopulmonary Resuscitation

**DOI:** 10.7759/cureus.102814

**Published:** 2026-02-02

**Authors:** Akihito Deguchi, Hirohisa Fujikawa, Kazuaki Ueki, Chihiro Hirata, Michihiro Tamai

**Affiliations:** 1 Department of Internal Medicine, Suwa Central Hospital, Nagano, JPN; 2 Department of General Medicine, Juntendo University Faculty of Medicine, Tokyo, JPN; 3 Department of Emergency and General Internal Medicine, Fujita Health University Hospital, Aichi, JPN; 4 Department of Internal Medicine, Hyogo Prefectural Tamba Medical Center, Hyogo, JPN

**Keywords:** acute myocardial infarction (ami), anticoagulation therapy, chest compression, dual antiplatelet therapy (dapt), extracorporeal cardiopulmonary resuscitation (e-cpr), resuscitation complications, transcatheter arterial embolization (tae), traumatic liver injury (tli)

## Abstract

Although chest compressions are a crucial component of cardiopulmonary resuscitation (CPR), they can sometimes result in traumatic complications. The incidence of hepatic injury, although rare relative to rib and sternal fractures, may result in severe hemorrhage and may be fatal, especially in patients receiving concomitant antiplatelet and anticoagulant therapy. This risk is particularly pronounced after extracorporeal CPR (E-CPR), as dual antiplatelet therapy (DAPT) for acute coronary syndrome and unfractionated heparin for preventing circuit thrombosis are required. We present a case of traumatic liver injury (TILI) following E-CPR for refractory ventricular fibrillation (VF) owing to acute myocardial infarction (AMI). In the absence of contrast extravasation on contrast-enhanced computed tomography (CT), nonoperative management was initially selected. However, the patient became transfusion-dependent despite receiving conservative management, prompting transcatheter arterial embolization (TAE), which resulted in rapid hemodynamic stabilization. The patient survived with a favorable neurological outcome. The present case highlights the need for maintaining a high index of suspicion for occult bleeding after E-CPR and underpins early minimally invasive hemostatic interventions, even in the absence of radiographic extravasation.

## Introduction

Cardiopulmonary resuscitation (CPR) is an essential life-saving intervention for patients experiencing cardiac arrest, aiming to maintain systemic circulation and oxygen delivery through chest compressions and ventilation. Cardiac arrest is frequently caused by lethal arrhythmias, such as ventricular fibrillation (VF), during acute myocardial infarction (AMI). In such situations, immediate CPR has been shown to improve survival rates [1].

Extracorporeal CPR (E-CPR) is an emergency procedure utilizing veno-arterial extracorporeal membrane oxygenation (VA-ECMO) for patients with cardiac arrest when conventional CPR fails. Early initiation of E-CPR for refractory VF has been reported to markedly improve survival to hospital discharge [2].

Although effective CPR requires forceful chest compressions, it can result in traumatic complications, including skeletal fractures and internal organ injuries. Rib and sternal fractures are the most frequently reported injuries; however, visceral organ damage involving the liver, lungs, heart, and spleen has also been described. Among CPR-related traumatic complications, hepatic injury is rare, with an incidence of 0.6-2.1% [3,4]. Despite its low incidence, hepatic injury can be life-threatening and therefore requires early recognition and appropriate management.

Traumatic liver injury (TLI) is most commonly caused by blunt trauma. Contrast-enhanced computed tomography (CT) is the key to its diagnosis, allowing assessment of injury severity and presence of active bleeding. Contrast extravasation on CT is a crucial indicator for therapeutic decision-making, and in its absence, careful nonoperative management is often desired [5]. However, the detection of extravasation requires a certain bleeding rate, and slow bleeding from microvascular injury or subcapsular hematomas may not be visualized on initial imaging. Consequently, TLI without any apparent extravasation on initial CT may still worsen over time.

Furthermore, patients receiving E-CPR for AMI are at increased risk of bleeding owing to the need for dual antiplatelet therapy (DAPT) and anticoagulation during VA-ECMO. Under these conditions, even minor liver injuries without clear radiographic findings may result in delayed and clinically significant hemorrhage.

The present report describes a case of TLI following E-CPR and percutaneous coronary intervention (PCI) for AMI with refractory VF. Despite the absence of extravasation on contrast-enhanced CT or angiography, the patient required blood transfusions and treatment with inotropic agents. Empiric transcatheter arterial embolization (TAE) successfully stabilized the patient's hemodynamics, ultimately resulting in a favorable neurological outcome.

## Case presentation

A previously healthy 50-year-old female patient was transported to the emergency department because of acute chest pain. She was initially alert and oriented on arrival but experienced sudden cardiac arrest shortly thereafter, with VF as the presenting rhythm. CPR was immediately initiated. Despite three defibrillation attempts and administration of epinephrine (1 mg) and amiodarone (300 mg), return of spontaneous circulation (ROSC) was not initially achieved. The patient was intubated, and VA-ECMO access was established. After 13 minutes of CPR, ROSC was finally attained. A fourth defibrillation successfully terminated VF. Electrocardiogram (ECG) demonstrated ST-segment elevations in the inferior leads, raising concern for acute ST-elevation myocardial infarction.

**Figure 1:**
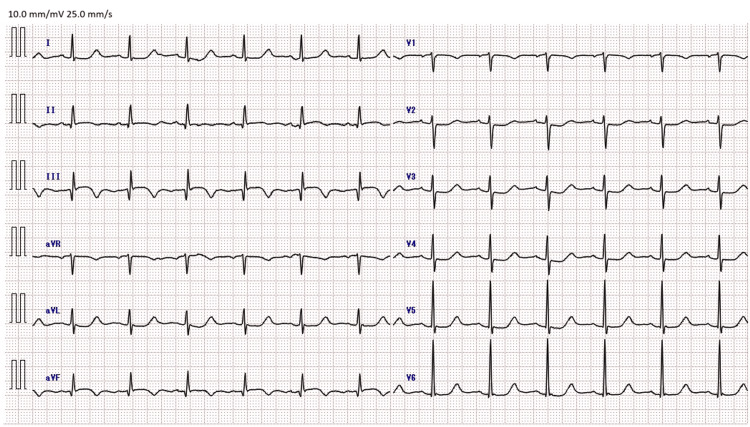
Electrocardiogram at the time of admission to the intensive care unit

Emergency coronary angiography revealed occlusion of the proximal right coronary artery. Loading doses of aspirin (200 mg) and prasugrel (20 mg) were administered, followed by primary PCI along with placement of a drug-eluting stent. For VA-ECMO management, unfractionated heparin was administered and titrated to maintain an activated partial thromboplastin time of 50-70 seconds. The patient was then admitted to the intensive care unit. ECG showed resolution of ST-segment elevation in the inferior leads with newly developed negative T waves, consistent with successful reperfusion (Figure 1).

On the first day of hospitalization, the patient received transfusions of four units of packed red blood cells (RBCs) and four units of fresh frozen plasma (FFP) to maintain adequate VA-ECMO flow. Although vasopressor support was not initially required, the patient had gradually worsening hypotension approximately four hours later, necessitating vasopressor initiation. On the second day, at 13 hours post admission, she required 0.2 μg/kg/min norepinephrine and developed marked anemia, with the hemoglobin levels declining from 13.3 to 7.2 g/dL (Table 1). Abdominal ultrasonography revealed a mass-like lesion on the liver surface and the presence of intraperitoneal fluid, indicating intra-abdominal bleeding. An additional six units of RBCs were subsequently administered.

**Table 1:** Laboratory test results

Variable	Day 1	Day 2	Day 29	Unit	Reference range
Arterial blood gas					
pH	7.148	7.56		-	7.37–7.44
pCO_2_	26.3	38.1		mmHg	34-45
pO_2_	496.4	85.9		mmHg	70-90
Complete blood count					
White blood cells	17960	8250	5550	/μL	3500–9100
Red blood cells	414	236	331	×10^4^/μL	380–480
Hemoglobin	13.3	7.2	10.4	g/dL	11.3–15.2
Platelets	25.6	6.1	28.3	×10^4^/μL	13–36.9
Electrolytes					
Sodium	142	143.3	136.5	mEq/L	138–145
Potassium	4	4.7	4.7	mEq/L	3.6–4.8
Chloride	111	112	103	mEq/L	101–108
Calcium	9.4	7.4		mg/dL	8.8–10.6
Inorganic phosphorus	3.3	5.8		mg/dL	2.7–4.6
Proteins, enzymes, and others					
Blood urea nitrogen	16.7	19.3	13.1	mg/dL	8–20
Creatinine	0.97	1.17	0.8	mg/dL	0.6–1.1
Uric acid		4.4		mg/dL	2.0–7.0
Total bilirubin	0.15	0.89	0.8	mg/dL	0.4–1.5
Aspartate aminotransferase	33	240	19	IU/L	<30
Alanine aminotransferase	14	136	20	IU/L	<30
Alkaline phosphatase	162	79	173	U/L	38–113
Lactate dehydrogenase	366	710	386	IU/L	124–222
Gamma-glutamyl transpeptidase	111	94	76	U/L	0–64
Total protein	7	4.2	7.6	g/dL	6.7–8.3
Albumin	3.6	2.5	3.5	g/dL	3.9–4.9
Blood glucose	99			mg/dL	74–109
Creatine kinase	117	1734	9	IU/L	0–170
Troponin I	1.841			ng/mL	<0.030
B-type natriuretic peptide	81.7			pg/mL	<18.4
C-reactive protein	5.09	4.47		mg/dL	<0.3
Lactate	11.24	1.79		mmol/L	
Coagulation					
Activated partial thromboplastin time	29.6	60.1		sec	24–34
Prothrombin time	11.7	18		sec	11–14
Prothrombin time international normalized ratio	1.02	1.56		-	0.91–1.12
Fibrinogen	330	261.2		mg/dL	200–400
D-dimer	0.8	31.8		μg/mL	<1.0

**Figure 2:**
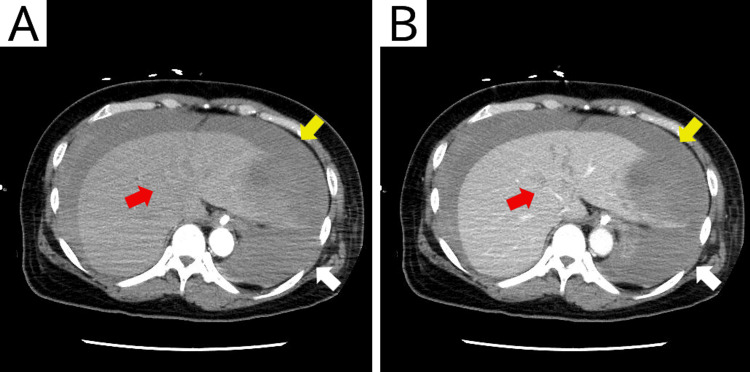
Contrast-enhanced computed tomography of the abdomen (A: arterial phase; B: portal venous phase) shows hepatic lacerations (red arrow) with associated hemoperitoneum (white arrow) and hepatic hematoma, including subcapsular and intraparenchymal components (yellow arrow). The images reveal no contrast extravasation.

As the patient’s native cardiac output had recovered, VA-ECMO was removed percutaneously with balloon hemostasis, and anticoagulation therapy was discontinued. DAPT, comprising maintenance-dose aspirin (100 mg/day) and prasugrel (3.75 mg/day), was continued because of the high risk of coronary stent thrombosis in the patient. Contrast-enhanced CT revealed an American Association for the Surgery of Trauma Grade IV liver injury, characterized by large liver lacerations, subhepatic hematoma, and hemoperitoneum (Figure 2). No contrast extravasation or pseudoaneurysm was observed; thus, active bleeding was considered unlikely. The intra-abdominal pressure, measured using a bladder catheter, was 10 mmHg, with no evidence of abdominal compartment syndrome.

**Figure 3:**
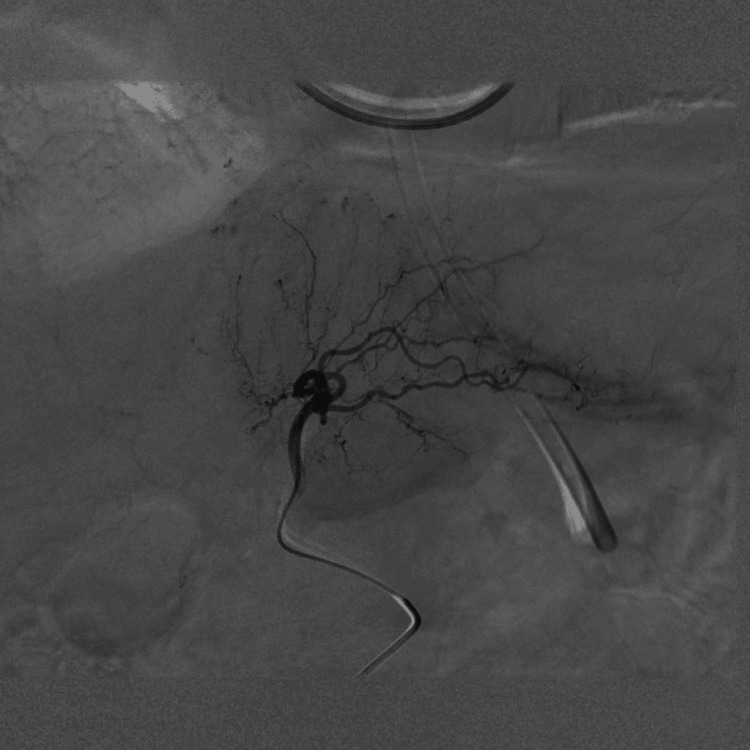
Angiography showing no contrast extravasation

Nonoperative management was initially selected to prevent the risk induced by operation, absence of contrast extravasation, and lack of evidence for abdominal compartment syndrome. Despite aggressive volume resuscitation with additional transfusions of eight units of RBCs, 10 units of FFP, and 20 units of platelets, the patient remained transfusion-dependent and required escalating vasopressor support. Her blood pressure partially responded to volume loading; however, norepinephrine alone was insufficient, and 0.03 U/min vasopressin was added. Given the persistent hemodynamic instability, TAE was performed. Angiography demonstrated no contrast extravasation (Figure 3); nevertheless, embolization of the left hepatic artery branch using gelatin sponge particles stabilized the patient’s hemodynamics, allowing rapid tapering and early discontinuation of vasopressors, including norepinephrine and vasopressin.

On the third day of hospitalization, the hemoglobin level decreased to 6.3 g/dL, necessitating transfusion of six units of RBCs; however, vasopressor support was no longer required. On the fifth day, the patient was extubated. Her hemoglobin level was 7.4 g/dL, and two additional units of RBCs were transfused without further hemodynamic deterioration. A follow-up non-contrast-enhanced CT performed on the fifth day revealed a new low-density lesion in the posterior segment of the right hepatic lobe, suggestive of an additional liver hematoma. Despite this finding, the patient’s hemodynamics remained stable, and the volume of ascites had decreased; therefore, close observation was continued.

The patient demonstrated a positive recovery trajectory, with the hemoglobin level increasing to 10.4 g/dL. Follow-up contrast-enhanced CT on the 14^th^ day of admission demonstrated interval reduction of hematomas in both hepatic lobes, confirming favorable radiologic progression.

The patient was discharged on the 30^th^ day of hospitalization following rehabilitation, walking independently and without any neurological deficits (Cerebral Performance Category 1). A three-month follow-up contrast-enhanced CT scan demonstrated gradual resolution of the hepatic hematomas.

## Discussion

This paper describes a rare case of delayed hemorrhagic shock caused by TLI after E-CPR. Notably, neither contrast-enhanced CT nor angiography demonstrated contrast extravasation; however, TAE successfully achieved hemodynamic stabilization and prevented further progression of anemia. Ten articles have been published on TLI after E-CPR (Table 2) [6-15]. The present case highlights the need for prompt recognition and timely intervention for TLI in patients undergoing DAPT and anticoagulation therapy, even in the absence of radiographic evidence for active bleeding.

**Table 2:** Previous cases of traumatic liver injury secondary to extracorporeal cardiopulmonary resuscitation DCS: damage control surgery; NOM: non-operative management; PTE: pulmonary thromboembolism; VF: ventricular fibrillation; TAE: transcatheter arterial embolization; NA: not available; CPC: Cerebral Performance Category

Case no. [Ref]	Year	Age	Sex	Diagnosis	Liver injury location	Active extravasation	Management	Neurological outcome
1 [6]	2012	50	Male	VF	Right lobe	+	DCS	Survived (neurological outcome was bad, but CPC was not reported)
2 [7]	2014	34	Male	PTE	Both lobe	+	DCS	Good (CPC was not reported)
3 [8]	2017	N.A.	N.A.	VF	Left lobe	N.A.	TAE	Good (CPC not reported)
4 [9]	2017	78	Female	PTE	Right lobe	+	NOM	Died
5 [9]	2017	81	Female	PTE	Right lobe	+	DCS	Survived (neurological outcome not reported)
6 [10]	2018	61	Female	PTE	Left lobe	+	DCS	Survived (neurological outcome not reported)
7 [11]	2020	57	Female	PTE	Left lobe	-	NOM	Died
8 [11]	2020	70	Male	VF	Left lobe	-	NOM→DCS	Good (CPC not reported)
9 [12]	2020	70	Male	VF	Left lobe	-	NOM→DCS	Good (CPC not reported)
10 [13]	2022	53	Female	VF	Left lobe	+	DCS	Survived (neurological outcome not reported)
11 [14]	2023	62	Male	VF	Left lobe	-	NOM→DCS	Survived (neurological outcome not reported)
12 [14]	2023	63	Male	VF	Left lobe	+	DCS→TAE	Survived (neurological outcome not reported)
13 [15]	2024	33	Female	VF	Left lobe	+	DCS	Survived (neurological outcome not reported)
Present case	2025	50	Female	VF	Left lobe	-	NOM→TAE	Good (CPC 1)

Although hepatic injury after CPR is uncommon, it can be fatal once it occurs [9,16]. Skeletal injuries are frequent, whereas visceral organ damage is relatively rare [17]. The left hepatic lobe is particularly vulnerable because of its close anatomical proximity to the xiphoid process, rendering it susceptible to compression-associated injuries [10].

Imaging plays a central role in the diagnosis of hepatic injury, with abdominal ultrasonography and contrast-enhanced CT being the most widely used modalities. Ultrasonography enables rapid bedside assessment, whereas contrast-enhanced CT provides detailed evaluation of the severity and extent of injury [18]. Although contrast extravasation is an important indicator of active bleeding, its detection requires a sufficiently high bleeding rate, and microvascular hemorrhage may fall below the detection threshold [6]. In patients receiving antiplatelet and anticoagulant therapies, even minimal bleeding can progress and become life-threatening; this risk increases further when DAPT is combined with anticoagulation therapy [12].

The treatment options for hepatic injury include TAE, laparoscopic surgery, and damage control surgery. Surgical intervention is highly invasive and poses a substantial risk in patients with shock receiving DAPT and anticoagulation. In contrast, TAE is a minimally invasive option and can provide rapid hemostasis, making it an effective option when surgery is not feasible [19,20]. Careful monitoring of hemodynamics and hemoglobin level changes is essential to determine the optimal timing of intervention. In the present case, persistent hemodynamic instability and progressive anemia, despite transfusion support, were considered critical decision thresholds for intervention, even in the absence of radiographic evidence of active bleeding. TAE was thus performed, which achieved effective hemostasis.

Furthermore, follow-up imaging revealed a new subcapsular hematoma in the right hepatic lobe that resolved spontaneously despite continued DAPT, without any hemodynamic compromise or worsening anemia. This finding suggests that TLI may worsen over time under antithrombotic therapy; however, hemostatic intervention may not be required in the absence of clinical deterioration.

## Conclusions

TLI caused by CPR is a rare but potentially fatal complication, particularly in patients receiving antiplatelet and anticoagulant therapies. Even in the absence of contrast extravasation, hemostatic intervention should be considered for hemodynamic instability that persists. In such situations, surgical hemostasis is often impractical, and TAE represents an effective alternative. The present case highlights the importance of maintaining vigilance for occult bleeding and implementing prompt, imaging-guided, multidisciplinary interventions in the management of patients following E-CPR.
